# Hybrid Nanoassemblies from Viruses and DNA Nanostructures

**DOI:** 10.3390/nano11061413

**Published:** 2021-05-27

**Authors:** Sofia Ojasalo, Petteri Piskunen, Boxuan Shen, Mauri A. Kostiainen, Veikko Linko

**Affiliations:** 1 Biohybrid Materials, Department of Bioproducts and Biosystems, Aalto University, P.O. Box 16100, 00076 Aalto, Finland; 2 Department of Medical Biochemistry and Biophysics, Karolinska Institutet, 17165 Stockholm, Sweden; 3 HYBER Centre, Department of Applied Physics, Aalto University, P.O. Box 15100, 00076 Aalto, Finland

**Keywords:** DNA nanotechnology, DNA origami, virus, capsid, protein, nanofabrication, biomedicine, self-assembly, vaccine

## Abstract

Viruses are among the most intriguing nanostructures found in nature. Their atomically precise shapes and unique biological properties, especially in protecting and transferring genetic information, have enabled a plethora of biomedical applications. On the other hand, structural DNA nanotechnology has recently emerged as a highly useful tool to create programmable nanoscale structures. They can be extended to user defined devices to exhibit a wide range of static, as well as dynamic functions. In this review, we feature the recent development of virus-DNA hybrid materials. Such structures exhibit the best features of both worlds by combining the biological properties of viruses with the highly controlled assembly properties of DNA. We present how the DNA shapes can act as “structured” genomic material and direct the formation of virus capsid proteins or be encapsulated inside symmetrical capsids. Tobacco mosaic virus-DNA hybrids are discussed as the examples of dynamic systems and directed formation of conjugates. Finally, we highlight virus-mimicking approaches based on lipid- and protein-coated DNA structures that may elicit enhanced stability, immunocompatibility and delivery properties. This development also paves the way for DNA-based vaccines as the programmable nano-objects can be used for controlling immune cell activation.

## 1. Introduction

**Figure 1:**
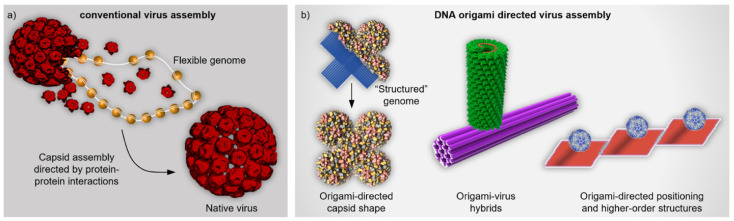
Comparison between conventional and DNA origami directed virus assembly. (**a**) Conventional virus assembly. Capsid proteins (CP, red) assemble into a complete native virus capsid through protein-protein interactions and simultaneously package the flexible genome (DNA or RNA) into it [39]. (**b**) DNA origami directed virus assemblies. Left: Virus capsid takes a non-native shape as the viral CPs assemble on top of the templating DNA origami that serves as “structured” genomic material similarly as in Ref. [40]. Middle: Tobacco mosaic virus (TMV) capsid assembles at the specific location of the DNA origami template. The capsid forms around the protruding RNA strand that contains a characteristic loop as an origin-of-assembly sequence [41]. Right: Virus capsids are bound to DNA origami platforms which are further assembled into higher-order structures [42]. (**a**) reproduced with permission from [39]. Copyright Royal Society of Chemistry, 2020. (**b**) Middle panel reproduced with permission from [41]. Copyright American Chemical Society, 2018. Right panel reproduced with permission from [42]. Copyright American Chemical Society, 2010.

One of the major branches in the bionanotechnology field searches for innovative strategies to be used in medicine and therapeutics. Long-term goals include modern drug and vaccine development, the creation of programmable nanocarriers with targeted delivery of therapeutic cargoes, and the fabrication of nanoscale devices for diagnostics. It becomes notoriously difficult to create such minuscule and multifunctional apparatuses from the top-down, and thus the groundbreaking solutions are often found from the realm of molecular self-assembly [1]. For example, native viruses and virus capsid proteins (CPs) are inherently functional and atomically precise, and the full viral capsid forms through CP-CP interactions, thus packaging the viral genome (DNA or RNA) into it [2] (Figure 1a). These versatile properties can be harnessed in creating multi-purpose biotemplates for various uses [2]. On the other hand, besides the naturally occurring family of viruses and proteins, there is a legion of artificial and customizable ångström-scale accurate nano-objects, such as de novo-designed proteins [3] and DNA nanostructures [4]. Arguably, the former is hugely important, but their design requires massive computational power. Therefore, in this review, the latter are considered, as DNA nanotechnology provides an easily accessible route to nanofabrication [5], and the custom DNA shapes can act as “structured” genomic material for a variety of virus assemblies (Figure 1b). Indeed, the coupling of viruses and DNA nano-objects may yield novel hybrid structures with intriguing user-defined features, described later in this review.

Viral proteins and DNA molecules have also proven to be highly controllable and programmable in a synthetic environment. These structures can be modified using various techniques, which makes both viruses and DNA applicable nanomaterials for functional nanoassemblies. Eminent examples of commonly employed viruses in nanotechnology are various plant viruses [6,7], such as *tobacco mosaic virus* (TMV) and *cowpea chlorotic mottle virus* (CCMV), whereas lattice-constructed MDa-scale ‘DNA origami’ [8,9] with nanoscale dimensions represent the most frequently used artificial DNA objects. DNA origami is based on folding a long single-stranded DNA into virtually any shape, geometry and topology with the help of dozens of short staple strands [5,8,9,10], and currently there exist extensive primers for designing and creating custom structures [11,12]. Recently, DNA nanostructure assembly toolbox has been expanded with wireframe structures [13,14], automated design methods [15,16], GDa-scale objects [17], micrometer-scale fractal assemblies [18] and constructs with up to 10,000 individual and unique strand components [19], thus lowering the barriers for synthesizing objects at the size range of viruses and cellular organelles. Besides the discrete objects, well-ordered DNA origami-based 2D lattices can reach ~10 cm^2^ surface areas [20] and 3D DNA crystals millimeter scale dimensions [21,22]. So far, these versatile DNA nanoshapes have found applications in biomedicine, diagnostics and therapeutics [23,24], nanofabrication [25,26], molecular electronics [27,28] and super-resolution imaging [29], and as nanorulers [30], plasmonic or photonic apparatuses [31,32], precise nanoscopic measurement tools [33], tunable nanopores [34,35] and dynamic/robotic devices [36,37].

This development has also enabled a number of DNA-protein assemblies [38], and a handful of rather innovative examples of hybrid DNA origami-viral protein-complexes. These complexes have several properties that make them promising tools for prospective bionanotechnology applications. These hybrids can be considered biocompatible, often non-toxic, and their structural features can be harnessed in smart targeted delivery, and they may be able to penetrate in vivo barriers. The structural programmability provides a plethora of possibilities in designing structures for specific, even dynamic functions. When considering the cellular intake, the CPs and other proteins with virus-mimicking properties are mainly employed for enhanced transfection efficiency, whereas the DNA nanostructures are used for multiple cargo carriers such as drug molecules, imaging rea-gents or gene transfers. In addition, several research groups have experimented with coating and decorating DNA origami with viral capsid proteins (CPs), employing various shapes of origami and different viruses.

In this concise review, we cover the existing literature on DNA nanostructure-virus hybrids. In Section 2, we discuss how different factors such as the shape of the DNA origami affect the encapsulation into CCMV and simian virus 40 (SV40) capsids (Figure 1b, left), and furthermore, how the encapsulation enhances the cellular delivery of DNA origami. Moreover, we cover in situ and dynamic TMV assemblies on RNA-functionalized DNA origami (Figure 1b, middle) and present some basics of virus-DNA origami coupling (Figure 1b, right). Section 3 focuses on the virus-mimicking approaches, such as lipid, protein and peptoid coatings of DNA nanostructures and discusses the possibility to develop DNA origami-based vaccines through programmable antigen-triggered B-cell activation. In the Conclusion, we briefly introduce some other virus-DNA-related implementations and provide a summary table of the currently available hybrids, their assembly and characterization methods and their potential applications.

## 2. Hybrid Nanoassemblies from Viruses and DNA Motifs

As explained above, virus capsids could serve as accurate components for versatile molecular patterning and templates for e.g., dyes, catalysts, inorganic nanoparticles, polymers and drugs [2,42]. One of the first demonstrations to expand the patterning abilities to larger-length scales using DNA was presented by Stephanopoulos et al. [42]. In this work, the authors formed hybrid structures by attaching a poly-T-decorated bacteriophage MS2 capsid (diameter of ~27 nm) to a ssDNA-modified DNA origami platform through hybridization and verified the assembly using atomic force microscopy (AFM) and transmission electron microscopy (TEM). The work made use of triangular DNA origami and various rectangular DNA origami tiles that could be conjugated together, thus forming one-dimensional arrays of the capsids (Figure 1b, right). Moreover, as an example of cargo loading, the authors used the interior of the capsid to pack fluorescent dyes (Oregon Green maleimide) into it, however, functionality was not further shown. Regardless of the molecular cargo functionality, this research subtly demonstrated the capability of building higher order structures using DNA and viruses, and therefore bridging atomically precise features to micrometer length scales for arrays of over 10 conjugated tiles. Therefore, this pioneering research paved a way for further functional assemblies and applications introduced in this section.

### 2.1. Packaging DNA Origami into Viral Capsids

Mikkilä et al. [40] coated DNA origami with CCMV CPs via electrostatic interactions and demonstrated their enhanced cellular intake. The study focuses on the binding of CPs with the DNA origami and how the binding ratio *γ* (defined as by the number of CPs divided by the number of DNA base pairs in the sample solution) affects the transfection efficiency and the shape of the nanoassembly. These discoveries on transfection efficiency are especially interesting from the viewpoint of nanomedicine and nanoimaging.

The DNA origami used in the study was a 2D rectangular sheet [8] and the CPs for the coating were derived from CCMV, which has shown great capacity to accept synthetic molecules and protein guest molecules inside its capsid [6]. The CPs were purified from the native virus and they held their positive charge in the N-terminus. This important feature allows the CPs to bind and self-assemble on the negatively charged DNA origami.

Binding of the CPs was studied using an electrophoretic mobility shift assay (EMSA). A decrease in electrophoretic mobility of the DNA origami-CP complex was observed as the value of *γ* increased, thus indicating efficient binding of CPs to the DNA origami.

**Figure 2:**
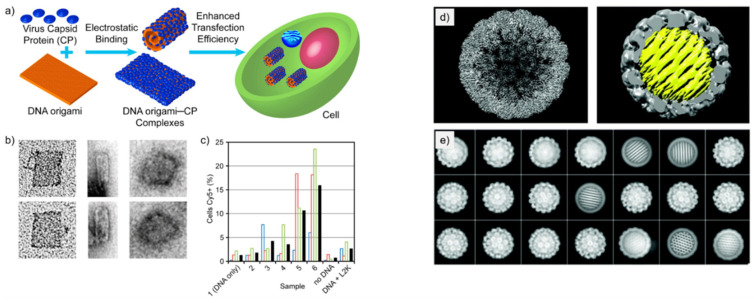
DNA origami encapsulation inside virus capsid proteins. (**a**) Schematic illustration of the formation of DNA-origami-CCMV CP complexes via electrostatic interactions and enhanced transfection to cells [40]. (**b**) TEM images of DNA origami-CP complexes with various *γ* values (from left to right: *γ* = 0, *γ* = 0.08, *γ* = 0.64). (**c**) Quantification of DNA origami–CP positive cells with HCS microscopy (*γ* increases gradually from samples 1 to 6). Colored open bars indicate measurements from individual samples; black-filled bars are calculated mean values of the triplicate samples. (**d**) Reconstructed SV40 particles: left, empty capsid; right, SV40 capsid (gray) encapsulated spherical DNA origami (yellow) of 35 diameter [44]. (**e**) Class averaged particles images show both the empty capsids and the encapsulated DNA origami in different orientations. (**a**–**c**) reproduced with permission from [40]. Copyright American Chemical Society, 2014. (**d**,**e**) reproduced with permission from [44]. Copyright Royal Society of Chemistry, 2019.

The morphology of the DNA origami-CP complexes was visualized using TEM with the same *γ* values as in EMSA. TEM imaging showed that at *γ* = 0.08 the DNA origami-CP complexes started to bend in tube-like conformations, while at the ratio of *γ* = 0 the DNA origami were observed in their rectangular shape (Figure 2a,b). Half-bent structures of DNA origami were observed as well. With the value of *γ* = 0.64 (Figure 2b), the DNA origami-CP complexes were observed as round rather than tube-like. The changes in the structure of DNA origami after adding CPs are plausibly happening due to electrostatic effects. The positively charged CPs most probably reduce the repulsion between the adjacent DNA helices with a negative charge. It was proposed that these observations indicate the complete encapsulation of DNA origami.

Transfection efficiency of DNA origami-CP complexes was investigated as well. Samples with a higher value of *γ* showed increased aggregates in high content screening microscopy of the cells that had been incubated by DNA origami-CP complexes. Sample with *γ* = 0.64 showed a 13-fold enhancement of entering cells compared to a DNA origami sample that had no CPs bound to it. This indicates that coating DNA origami with CCMV CPs significantly enhances their transfection efficiency. The detailed protocol of the encapsulation is provided by Linko et al. in Ref. [43].

Kopatz et al. [44] studied the packaging of a nearly spherical DNA origami with simian virus 40 (SV40) CPs called VP1. The aim of the study was to test how well the DNA origami works as a substrate for the self-assembly of SV40 CPs, and whether it can be fully and efficiently encapsulated by the CPs. Nucleic acids have limited flexibility and packing density, and therefore the encapsulation process is difficult when the nucleic acid molecule does not match with the native viral genome. However, DNA origami as a “structured” genome provides a solution for this, and it is therefore used in this study.

Three spherical DNA origami designs varying in diameter were employed in the study. All the three different DNA origami had the same honeycomb structure and were densely packed. The diameters of the studied DNA origami were 30, 35, and 40 nm, and these sizes were chosen to resemble the size of the cavity in the native SV40. DNA origami with a 40-nm diameter formed larger particles together with the capsid when compared to the native SV40, while DNA origami with a 30-nm diameter yielded particles that were about the right size, however they came with some irregularities, such as with incomplete coatings and oval-shaped particles. The DNA origami with a 35-nm diameter was chosen as the assembly substrate of the SV40 CPs, as together with the capsid, the particle had a 50-nm diameter, which corresponds the one of the native SV40.

The DNA origami-CP particles were analyzed using TEM, agarose gel electrophoresis (AGE) and cryo-electron microscopy (cryo-EM) single particle analysis. TEM imaging showed highly uniform and fully coated DNA origami-CP complexes alongside VP1 particles and pure DNA origami in a solution that had a molar ratio of 400 VP1 pentamers per DNA origami. No partly encapsulated DNA origami were found, indicating that the assembly process is strongly cooperative. However, empty icosahedral viral capsids encapsulating no DNA origami were observed, which might be explained by some DNA origami remaining unfolded. A 54% yield of encapsulated DNA origami was achieved. The reformed capsid had an icosahedral symmetry (capsid triangulation number *T* = 7d), which corresponds to the native SV40 capsid structure. The honeycomb DNA origami was regularly positioned inside the capsid so that the DNA helices were tilted at a ~6.8° angle in relation to the icosahedral three-fold symmetry axis of the capsid (Figure 2d). The successfully formed DNA origami-CP complexes using 35-nm honeycomb DNA origami and VP1-proteins of SV40 have a corresponding morphology to the native virus, therefore it is more likely to possess similar functional qualities as the native virus. This study illustrates yet another elegant approach to create biohybrid nanoassemblies with high fidelity.

### 2.2. In Situ and Dynamic Virus Assemblies on DNA Origami

**Figure 3:**
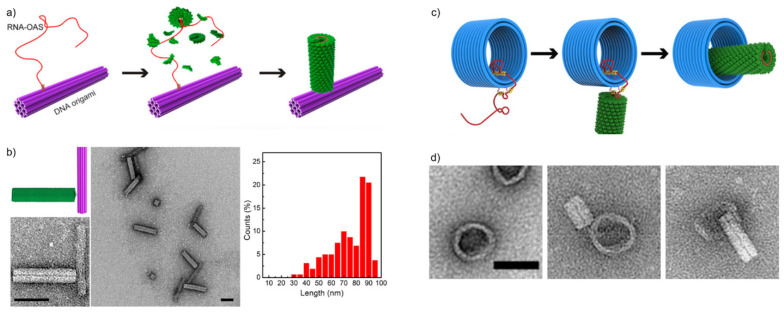
DNA origami-virus hybrids based on *Tobacco mosaic viruses* (TMV). (**a**) The in situ assembly process of TMV CPs around a TMV RNA strand anchored to a DNA origami platform [41]. The origin of assembly (OAS) sequence nucleates the growth of the nanotube. (**b**) Assembled TMV-DNA origami hybrids viewed with TEM. The graph on the right shows the length distribution of assembled tubes around a ~1910-nt long RNA. Scale bars are 50 nm. (**c**) The in situ assembly of a TMV rod nestled inside a DNA origami barrel [45]. The assembly is guided by pathing the RNA strand through multiple binding sites along the DNA origami and then releasing the bound RNA in a stepwise manner. The DNA origami barrel was a 23-helix bundle with a length of ~30 nm and an inner diameter of ~34 nm. (**d**) TEM images of the steps depicted in Figure 3c. Left: the DNA origami barrel with pathed RNA. Middle: CP rod assembly on the unbound overhang of the RNA. Right: A finished CP rod inside the hollow of a DNA origami barrel. Scale bar is 50 nm. (**a**,**b**) reproduced with permission from [41]. Copyright American Chemical Society, 2018. (**c**,**d**) reproduced with permission from [45]. Copyright American Chemical Society, 2020.

Zhou et al. [41] studied the in situ assembly of *tobacco mosaic viruses* (TMVs) onto DNA origami. The study exploited the natural tendency of viral proteins to assemble around their own genome, thus encapsulating their genetic material. The RNA genome (or a modified sequence) of a TMV contains a characteristic loop that serves as an origin-of-assembly sequence (OAS). The OAS motif first nucleates a double-layer disk composed of TMV subunits and the capsids keep piling tightly around the RNA, twisting into a hollow, helical cylinder (See Figure 3a). Zhou et al. demonstrated that these types of nanotubes could be assembled in situ in a controlled manner by immobilizing TMV genome-mimicking RNA strands on a DNA origami surface before capsid assembly. Their technique functioned in creating TMV-like CP nanotubes in well-defined assembly patterns where the length of the nanotubes is dependent on the length of the used RNA strands.

To accomplish this, they used a rod-shaped 24-helix bundle (24HB) DNA origami, 16 nm in width and 100 nm in length, as a folding platform for the in situ assembly process. A TMV mimicking RNA strand was then immobilized onto the DNA origami via Watson-Crick base-pairing before starting the self-assembly process of the CPs. To investigate how well the nanotubes would form, they used three different lengths of RNA: 720 nucleotides (nt) as short-length (SL), 1230 nt as medium-length (ML) and 1910 nt as long-length (LL). The study showed that it is possible to generate TMV-like nanotubes with theoretically predictable lengths as the observed nanotubes were all around 30 nm for the SL RNA, 54 nm for the ML RNA and 86 nm for the LL RNA. Most of the nanotubes were observed to vary around 10 nm in length. However, the length distribution of the long RNA strands (see Figure 3b) was greater than for their shorter counterparts. The authors suggest that this has to do with the degradation of RNA at longer lengths. Furthermore, the yields of these DNA origami-CP complexes decreased from ~76% using the SL RNA to ~68% using the ML RNA to ~56% using the LL RNA. The authors proposed that this happened likely due to larger electrostatic repulsion between the DNA origami and the longer RNA strands.

In the same study, Zhou et al. also created more intricate and programmed structures by changing the docking sites of the RNA strands on various DNA origami structures. By crafting two protein nanotubes on opposite ends of a 24HB DNA origami rod, they managed to form both centrosymmetric and asymmetric structures, where the lengths of the extending tubes were again defined by the lengths of the corresponding RNA strands. Besides 24HB, Zhou et al. also used a 2D triangle-shaped DNA origami and a 3D tripod-shaped DNA origami as assembly platforms. SL, ML and LL RNAs were positioned on each vertex of the 2D triangular DNA origami leading to the formation of five different two-dimensional DNA origami-CP structures. For the 3D structure, a maximum of three ~54-nm TMV nanotubes were formed on the vertex, but with only a ~11% yield. Finally, they also created a DNA origami-CP complex where three short ~30-nm nanotubes were attached to the ends of the three tripod arms, one for each.

By forming these different structures, Zhou and coworkers demonstrated that this kind of approach is useful for creating both two- and three-dimensional, highly controllable DNA origami-CP complexes. They showed that it is possible to create more intricate complexes by engineering both the number and location of the TMV RNA on the used DNA origami platforms. Furthermore, tailoring the lengths of the attached TMV nanotubes by altering the lengths of the used RNA strands could be used to add a further layer of complexity to creatable structures. This indicates the possibility of forming precise and highly programmable configurations of DNA origami-CP nanoassemblies.

To further expand on their earlier work, Zhou et al. [45] then investigated the dynamic assembly of the TMV capsid proteins, again exploiting TMV genome-mimicking RNA anchored on DNA origami. Instead of just determining an assembly location for the TMV capsids, they were now able to guide the forming of the nanotubes during the assembly process by placing “path-points” along the DNA origami platforms. These path-points are simply ssDNA strands that extend out of the DNA origami, and they are complementary to certain parts of the virus RNA sequence. The ssDNA strands anchor the respective parts of the RNA onto the DNA origami and thus function as a local block for the TMV assembly. By including a toehold region to the anchor strands, the RNA can then also be released stepwise from these hindrances via toehold-mediated strand displacement, which provides a further degree of control over the assembly process.

To demonstrate this, Zhou et al. used a three-dimensional triangular DNA origami and a hollow rod-shaped barrel DNA origami. The employed TMV genome-mimicking RNA strand was similar to the ML strand used in their earlier study [41], 1234-nt long and containing the assembly-triggering OAS. First, the RNA strand was anchored on the triangular DNA origami on its 3′-end by fully complementary 40-nt capture strands. The triangular DNA origami was also decorated with 17 pairs of DNA strands to route the RNA and enable the dynamic assembly of the CP rod. Each of these DNA strands were 29-nt long, and of those, 13 nucleotides were complementary to the routed RNA. The remaining 16 nt formed the toehold region for release strands.

The OAS triggered the self-assembly of TMV CPs around the RNA strand, and it continued until the rod reached the first lock-point of RNA. With the help of the stepwise addition of release-strands, the dynamic assembly proceeded onwards. Based on TEM visualizations, the lengths of the protein rod and RNA corresponded to the theoretical growth in each phase based on the length of the released RNA. The protein rod grew from 27 nm in length before none of the routed RNA was released, to 54 nm after the entire RNA had been released.

Next, they applied this mechanism to the barrel DNA origami in an attempt to arrange the TMV rod inside the barrel. At first two 40-nt DNA strands were attached to one end of the barrel to bind 80-nt in the 3′-end of the RNA. The interior of the barrel and the outer surface on the other end were then decorated with ssDNA path-points. The pathing was arranged to guide the assembly of the TMV rod inside the barrel. After the TMV subunits were added, a 24-nm long nanotube could be seen where the free overhang of the RNA was located. After releasing the RNA from the path-points inside the barrel, Zhou and colleagues managed to form a complete, 54-nm long TMV nanotube nested within the DNA origami barrel (see Figure 3c,d). This way Zhou et al. established that this kind of confined and stepwise pathing approach could be used to place self-assembled CP structures even into otherwise spatially hindered positions on DNA origami, expanding the scope of attainable architectures.

## 3. Virus-Mimicking Approaches Based on DNA Origami Platforms

### 3.1. Tailored Lipid Coatings

Many viruses are surrounded by continuous lipid bilayer membranes which protect the enclosed viral nucleocapsids, as well as facilitate the entry of the capsids into host cells. To achieve similar goals, DNA origami have also been coated by lipids with various approaches to mimic native viruses.

**Figure 4:**
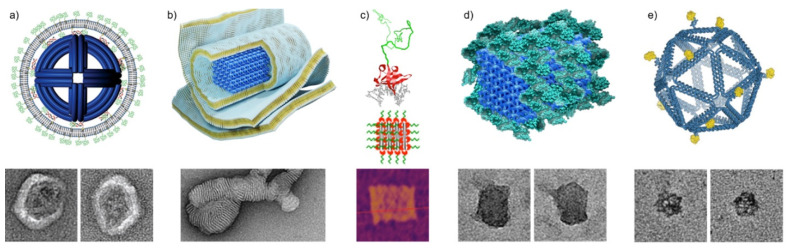
DNA origami directed assemblies mimicking virus structures. (**a**) Virus-inspired lipid membrane coating of DNA origami octahedron [46]. TEM insets are 100 nm × 100 nm. (**b**) Multilamellar lipid assemblies on 60HB DNA origami [47]. The TEM inset is 125 nm × 250 nm. (**c**) Modular protein polymer with a nonspecific DNA binding domain coating a DNA origami tile [51]. Green: C_8_ polypeptide bristle. Red: B^Sso7d^ binding domain. Gray: dsDNA. The AFM inset is 200 nm × 200 nm. (**d**) Inert bovine serum albumin coating of 60HB DNA origami [52]. The TEM insets are 80 nm × 80 nm. (**e**) Icosahedral wireframe DNA origami with programmable antigen patterns. The insets are 80 nm × 80 nm [58]. (**a**) reproduced with permission from [46] (https://pubs.acs.org/doi/10.1021/nn5011914 accessed on 26 May 2021). Further permissions related to the material excerpted should be directed to the American Chemical Society. Copyright American Chemical Society, 2014. (**b**) reproduced with permission from [47]. Copyright John Wiley and Sons, 2021. (**c**) reproduced with permission from [51]. Copyright American Chemical Society, 2017. (**d**) Reproduced with permission from [52]. Published by John Wiley and Sons, 2017. (**e**) reproduced with permission from [58]. Copyright Springer Nature, 2020.

Perrault and Shih [46] introduced virus-inspired membrane enveloped DNA nano-octahedrons (DNO) with decreased immune response and enhanced stability for biomedical applications. The DNO is a wireframe DNA origami with a diameter of ~50 nm decorated with fluorophore-conjugated oligos inside and lipid-conjugated oligos outside. The lipid-conjugated oligos function as anchors of the unilamellar envelope (Figure 4a). In addition to the main lipid component (DOPC, 94.2%), the membrane also comprises PEGylated lipid (PEG-DOPE, 5%) and fluorescent labelled lipid (Rh-DOPE) (0.8%) as fusion inhibitor and fluorescent marker, respectively. In vitro IL-6 and IL-12 immunoassays show that the encapsulated DNO (E-DNO) has an immune activation rate two orders of magnitude below controls. Furthermore, a splenocyte assay shows that nanostructure activation of, and uptake by, immune cells can be almost fully attenuated by encapsulation in the lipid membrane. In vivo pharmacokinetics analysis also indicates a factor of 17 improvement in bioavailability of the E-DNOs, as they are more stable than the uncoated DNOs.

DNA nanostructures can also bind to charged lipid molecules via electrostatic interactions. Julin et al. [47] complexed DNA origami together with cationic lipids into lipoplexes and characterized their self-assembly. Three DNA origami designs, 6-helix bundle (6HB), 60HB [48], and a nanoplate were assembled with cationic lipid molecules 1,2-dioleoyl-3-trimethylammonium-propane (DOTAP). The DOTAP binds to the negatively charged phosphate backbone of DNA molecules electrostatically and, in combination of hydrophobic interactions, results in a multilamellar lipoplex (Figure 4b). The complexation was carried out in deionized water to reduce the screening effect of counterions near the DNA nanostructures. The yield of complexation was initially characterized by electrophoretic mobility shift assay (EMSA) and TEM, while electron tomography (ET) and cryogenic TEM (cryo-TEM) were employed to study the morphology of the lipoplex in detail. Moreover, the phosphorus element was clearly detected in the multilamellar assemblies by energy-dispersive X-ray spectroscopy (EDS). The encapsulated DNA origami are much more resilient to the digestion of nuclease DNase I. This straightforward and scalable approach to protect DNA origami could potentially find use in nanomedicine and biology.

In contrast with mimicking native viruses to coat DNA origami in lipid membranes, rationally designed DNA nanostructures could also be used as a frame to shape the liposomes formed inside them. Both monodispersed spherical liposomes [49] and elongated tubular liposomes [50] have been assembled using DNA origami as a guide.

### 3.2. Dense Protein Coatings

As a logical extension to using coating approaches with pre-made virus capsid proteins, the ability to use similar, but more tailorable protein coating components for protecting and functionalizing DNA has also garnered attention in recent years. As a prime example, Hernandez-Garcia et al. [51] developed a generalized method for coating DNA structures with engineerable protein polymer bristles of precise amino acid sequence and length. Their modular de novo protein polymers could be attached to DNA through a non-electrostatic and nonspecific DNA binding domain (B^Sso7d^), while the other properties of the polymer could be separately influenced by the chosen bristle-elements (here hydrophilic C_8_) linked to the binding domain (Figure 4c). Upon mixing with various 1D DNA structures or a 2D DNA origami tile, the resulting polypeptides (C_8_–B^Sso7d^) formed bristle-like shells around the DNA objects increasing their mechanical stiffness and stability in solution, without disturbing the initial form of the coated structures. Furthermore, the protein coatings were semi-permeable to strong sequence specific binders, which meant the coated DNA structures could retain some of their functionality and enzymatic accessibility. The coatings were demonstrated to slow down digestion by DNase I enzymes 5-fold. In contrast to electrostatic binders, the non-electrostatic B^Sso7d^ targets DNA specifically, and thus also avoids issues with negatively charged surfaces and polyelectrolytes.

A similar approach was taken simultaneously by Auvinen et al. [52], who also used proteins with conjugated binding domains for coatings. In their approach, a synthetic dendron binding domain was employed to electrostatically bind two different proteins, either bovine serum albumin (BSA) (Figure 4d) or class II hydrophobin (HFBI), to three-dimensional 60 HB DNA origami structures. The protein-dendron conjugates consisted of a well-defined cationic second generation dendron with 27 protonatable amines linked to a protein via a cysteine-maleimide bond. Both BSA and HFBI yielded dense and uniform single-layer coatings for the DNA origami test structure. The BSA coating in particular was shown to significantly improve the nuclease digestion resistance of DNA origami against DNase I and to enhance transfection rate (by ~2.5 fold) into target cells in vitro, studied by fluorescence-activated cell sorting (FACS) and confocal microscopy. Notably, the BSA coating could also attenuate immune activation as observed by following interleukin 6 (IL-6) production of primary splenocytes isolated from mice. Compared to the semi-permeable coatings by Hernandez-Garcia et al. [51], the BSA-dendron coatings were more dense and effectively non-permeable to the digestive enzymes, providing efficient protection against degradation as a trade-off for accessibility.

Recently, also fully tailored coating elements have been synthesized. Wang et al. [53] designed various peptoid-based coating agents with different peptoid sequences (PE1-9) and studied their effects on dsDNA and an octahedral wireframe DNA structure, with simulations and experiments in various solution conditions. Attachment of the peptoids to the DNA frame was facilitated by controlled electrostatic binding. Each peptoid sequence was designed with cationic moieties (*N*-(2-aminoethyl)glycine, Nae) either in a “brush” (PE1-3) or a “block” (PE4-5) conformation, respectively corresponding to either parallel or orthogonal alignment of peptoids on the DNA frames. Two designs were used as controls by replacing the cationic monomers in a brush sequence (PE6) and a block sequence (PE7) with neutral ones. Lastly, designs PE8 & PE9 had alkyne-modified peptoids that were used to bind functional cargo molecules to the DNA frames. Upon mixing with the octahedral DNA frames, the peptoids were able to coat and maintain the shape of the frame below a N/P ratio threshold of 0.5, with higher ratios leading to aggregation, as observed by TEM imaging. Increasing the number of neutral (*N*-2-(2-(2-methoxyethoxy)ethoxy)ethylglycine, Nte) moieties in the peptoids was found to further improve the stability of the octahedra. However, the peptoids without any cationic Nae moieties at all (PE6 and PE7) showed no behavioral changes at different N/P ratios, hinting that the interactions between the peptoids and the DNA frames are determined mainly by the number and arrangement of the cationic segments.

Of the designs, only PE2 with a higher concentration of positive charges was able to retain the shape of the octahedra in lowered Mg^2+^ concentrations as observed with TEM, dynamic light scattering (DLS) and small-angle X-ray scattering (SAXS). However, all peptoid coatings were able to protect the DNA frames in low Mg^2+^ phosphate-buffered saline (PBS)-buffer, likely thanks to the compensating Na^+^ concentration in the PBS. Similarly, PE2 also conferred the most protection against DNase I degradation, while the digestion resistance was overall improved with all peptoid designs. The PE2 coated octahedra could also protect fluorescently labeled BSA cargo attached within the cavity of the frames from protease digestion, and on a similar line, also decrease the drug release rate of frames loaded with doxorubicin. Finally, Wang et al. additionally showed that by incorporating reactive target binding groups into the peptoid sequences, the peptoids could be used to decorate coated frames with functional molecules such as fluorescent dyes (Azide-Flour 288) or antibodies (Trastuzumab, Tz) for imaging and cell-targeting applications.

Lastly, it is worth mentioning that also other kinds of protein-DNA hybrids have been employed for various effects, in addition to the more virus-mimicking approaches described above. Their scope includes for example using human serum albumin (HSA) [54] for increased serum stability, cationic HSA [55] for facilitating electrostatic coating assembly, transferrin [56] for enhancing rectangular origami transfection, and spermidine [57] for enabling the electrotransfection of DNA structures.

### 3.3. Vaccine Development Using Selective Antigen Positioning

Besides the enhanced stability and immunocompatibility of the virus-mimics, the DNA nanostructures can be also harnessed in vaccine development. Veneziano et al. [58] studied the B-cell activation using DNA origami as a platform for the clinical vaccine immunogen eOD-GT8, which is an engineered outer domain of HIV-1 glycoprotein-120. The authors investigated how the antigen spacing, copy number and the rigidity and dimensionality of the scaffold affected the activation of B-cells in vitro. The platform DNA origami facilitated integration of 1–60 antigen copies with different spatial organizations.

In this study, two types of DNA origami platforms were used. One was an 80-nm long 6HB rod shape and the other was a wireframe-based icosahedron with a diameter of ~40 nm (see Figure 4d). Short ssDNA overhangs were used as anchors for complementary and synthetic peptide nucleic acid (PNA) strands that were previously coupled to eOD-GT8 antigens. This way the number of antigens and their spatial position could be precisely controlled (Figure 4e).

The activation of B-cells was observed by following the intracellular calcium signaling which expresses the full activation of the B-cell membrane-bound receptor IgM (IgM-BCR). The authors discovered that DNA origami with none or only one eOD-GT8 bound had no effect on activating the B-cells. The calcium signaling increased when DNA origami was equipped with two to five eOD-GT8, however, more than five antigens did not enhance the signaling. Importantly, the positioning of eOD-GT8 also played a role in the B-cell activation. For the icosahedral DNA origami, the optimal distance between eOD-GT8 particles was ~25–35 nm, and 80 nm for the rod DNA origami.

## 4. Discussion and Conclusions

To conclude, it can be stated that both DNA origami and viruses may serve as excellent materials to exploit in biomedicine. They each have a variety of qualities which make them superior to several inorganic nanomaterials in physiological conditions. DNA origami has proven to be a safe, biocompatible and non-cytotoxic material that can carry drugs, enzymes and other functional molecules and deliver those to target cells [23]. Viruses, in turn, are robust, precise, naturally at the nanoscale and have evolved in nature to penetrate inside cells. When the viruses are modified into VLPs, they lose their infectivity and therefore are safe to use in vivo as well. Biohybrid materials formed by the union of these viruses and DNA structures have the capability to revolutionize smart targeted drug delivery and could be employed in both therapeutics and diagnostics. A summary of the various selected virus-DNA nanostructure hybrids, virus-mimicking approaches, characterization techniques and possible target applications is provided in Table 1.

The hybrid complexes may also be used in a parallel fashion both in therapeutic and diagnostic applications. Recently, Kwon et al. [59] studied the possibility to use star-shaped DNA nanostructure equipped with ten aptamers targeting dengue envelope protein domain III (ED3). The exact shape of the DNA nanostructure was designed to bind to ED3, thus functioning as a virus inhibitor. On the other hand, modularity of the DNA shape opens up an option to employ it also as a simultaneous virus sensor/detector via the aptamer-protein binding.

Biomedicine is however not the only field where DNA nanostructures and VLPs could be exploited. Several studies in the fields of nanoelectronics, nanofabrication, molecular computing and chemical sensing have experimented with DNA origami and VLPs [5,23]. Therefore, investigating especially the various assembly strategies of hybrid structures can be widely beneficial. For example, it has been demonstrated that both DNA origami and TMV viruses can be used as templates for molecular lithography [60], indicating that even hybrid structures may be potentially used in versatile solid-state patterning at the nanoscale.

Most of the exciting opportunities in virus-DNA hybrids research are still unexplored, and several challenges concerning optimization for physiological conditions remain to be solved, especially in case of DNA nanostructures [61,62]. The most prominent of these are stability at low-cation conditions, resistance against nucleases, low pharmacokinetic availability, low cell uptake, possible inflammatory response and accurate loading and release of drug molecules [23,24,46,52,61,62,63,64,65,66,67,68,69,70,71]. Outstanding issues in virus-based nanocarriers are related to unknown adverse effects caused by the adaptive immunity against the viral proteins [72]. Similarly, as DNA origami designs are based on genomic scaffold strands, they may not be compatible with all medical treatments [23,73]. However, successful immunostimulative properties have been demonstrated both with CpG-laden bacteriophages [72] and DNA origami [74,75].

Still, these nanoassemblies are most definitely worth investigating due to their vast potential, and groundwork is indeed ongoing as shown in the featured studies. Furthermore, it has been recently estimated in a model [73] that DNA nanostructure-based treatments may become commercially viable, if DNA nano-objects can be produced at large scale and with high quality.

## Figures and Tables

**Table 1:** Selected virus and virus-mimicking protein-DNA nanostructure hybrids, their assembly methods, characterization techniques and possible target applications/functions.

Hybrid Composition	Assembly Method	Characterization Technique (s)	Target Application/Function
MS2 capsids + rectangular and triangular DNA origami [42]	ssDNA-modified capsids + a complementary ssDNA overhang protruding from DNA origami	AFM, TEM	higher-order assemblies of viruses through conjugation of programmable DNA origami platforms
CCMV CPs + rectangular DNA origami [40]	positively charged N-terminus of CP + negatively charged DNA origami	EMSA, TEM, confocal microscopy	virus-encapsulation significantly enhances the cellular delivery rate of DNA origami
SV40 CPs + variety of spherical DNA origami [44]	cooperative assembly of VP1s around DNA origami	AGE, TEM, cryo-TEM	investigating the effect of DNA origami of different sizes as a substrate for SV40 assembly
TMV CPs + various DNA origami [41,45]	in situ assembly of CPs around pre-bound RNA + toehold-operated hybridization sites on DNA origami	TEM, AFM, fluorescence assay	confined and programmable assembly, complex architectures
PEGylated lipid bilayers + DNA origami octahedron [46]	lipid-DNA conjugates anchored to DNA origami + liposome addition (and surfactant removal) for a fused lipid bilayer	TEM, IL-6 & IL-12 immunoassays, DNase I digestion assay, splenocyte activation assay, flow cytometry, confocal microscopy, fluorescence assay	mimicking lipid envelope of virus, enhancing stability and immunocompatibility
cationic multilamellar lipid bilayer + 6HB, 60HB and plate DNA origami [47]	electrostatic and hydrophobic interactions	AGE, TEM, cryo-TEM, ET, EDS	improves nuclease resistance, a route to form DNA-templated lipid assemblies
*de novo* protein polymer bristles + 1D or 2D DNA origami [51]	nonspecific and nonelectrostatic DNA binding domain in polymer bristle	AGE, AFM, fluorescence microscopy, DNase I digestion assay	dense bristle coating with modular bristles enables tuning of the stability, mechanical properties and surface chemistry of DNA structures while still retaining accessibility for strong binders
dendron-modified BSA and HFBI + 60HB DNA origami [52]	positively charged dendron as a synthetic DNA binding domain + negatively charged DNA origami surface	AGE, TEM, DNase I digestion assay, IL-6 immunoassay, confocal microscopy, FACS	BSA protein corona improves the nuclease resistance, immunocompatibility and cellular delivery of DNA origami
peptoids + octahedral wireframe DNA [53]	positively charged moieties in peptoid + negatively charged DNA frame	AGE, TEM, DLS, SAXS, fluorescence assay, molecular dynamics simulation, DNase I digestion assay, protease digestion assay, magnesium depletion assay	alignment of coating molecules, tunable stability, cell-targeting, display of functional molecules
antigens + icosahedral wireframe and rod-like DNA origami [58]	eOD-GT8 antigens coupled to synthetic PNA strands + complementary ssDNA overhangs protruding from DNA origami	AGE, TEM, intracellular calcium indicator dye assay	activation of B-cell membrane-bound receptor IgM, vaccine development

## References

[1] Whitesides G.M., Grzybowski B.A. (2002). Self-assembly at all scales. Science.

[2] Singh P., Gonzalez M.J., Manchester M. (2006). Viruses and their uses in nanotechnology. Drug Dev. Res..

[3] Huang P.-S., Boyken S.E., Baker D. (2016). The coming of age of de novo protein design. Nature.

[4] Jones M.R., Seeman N.C., Mirkin C.A. (2015). Programmable materials and the nature of the DNA bond. Science.

[5] Nummelin S., Kommeri J., Kostiainen M.A., Linko V. (2018). Evolution of structural DNA nanotechnology. Adv. Mater..

[6] Young M., Willits D., Uchida M., Douglas T. (2008). Plant viruses as biotemplates for materials and their use in nanotechnology. Annu. Rev. Phytopathol..

[7] Steinmetz N.F., Evans D.J. (2007). Utilisation of plant viruses in bionanotechnology. Org. Biomol. Chem..

[8] Rothemund P.W.K. (2006). Folding DNA to create nanoscale shapes and patterns. Nature.

[9] Douglas S.M., Dietz H., Liedl T., Högberg B., Graf F., Shih W.M. (2009). Self-assembly of DNA into nanoscale three-dimensional shapes. Nature.

[10] Hong F., Zhang F., Liu Y., Yan H. (2017). DNA origami: Scaffolds for creating higher order structures. Chem. Rev..

[11] Castro C.E., Kilchherr F., Kim D.-N., Shiao E.L., Wauer T., Wortmann P., Bathe M., Dietz H. (2011). A primer to scaffolded DNA origami. Nat. Methods.

[12] Dey S., Fan C., Gothelf K.V., Li J., Lin C., Liu L., Liu N., Nijenhuis M.D.A., Saccà B., Simmel F.C. (2021). DNA origami. Nat. Rev. Methods Primers.

[13] Benson E., Mohammed A., Gardell J., Masich S., Czeizler E., Orponen P., Högberg B. (2015). DNA rendering of polyhedral meshes at the nanoscale. Nature.

[14] Piskunen P., Nummelin S., Shen B., Kostiainen M.A., Linko V. (2020). Increasing complexity in wireframe DNA nanostructures. Molecules.

[15] Veneziano R., Ratanalert S., Zhang K., Zhang F., Yan H., Chiu W., Bathe M. (2016). Designer nanoscale DNA assemblies programmed from the top down. Science.

[16] Linko V., Kostiainen M.A. (2016). Automated design of DNA origami. Nat. Biotechnol..

[17] Wagenbauer K.F., Sigl C., Dietz H. (2017). Gigadalton-scale shape-programmable DNA assemblies. Nature.

[18] Tikhomirov G., Petersen P., Qian L. (2017). Fractal assembly of micrometre-scale DNA origami arrays with arbitrary patterns. Nature.

[19] Ong L.L., Hanikel N., Yaghi O.K., Grun C., Strauss M.T., Bron P., Lai-Kee-Him J., Schueder F., Wang B., Wang P. (2017). Programmable self-assembly of three-dimensional nanostructures from 10,000 unique components. Nature.

[20] Xin Y., Shen B., Kostiainen M.A., Grundmeier G., Castro M., Linko V., Keller A. (2021). Scaling up DNA origami lattice assembly. Chem. Eur. J..

[21] Zheng J., Birktoft J.J., Chen Y., Wang T., Sha R., Constantinou P.E., Ginell S.L., Mao C., Seeman N.C. (2009). From molecular to macroscopic via the rational design of a self-assembled 3D DNA crystal. Nature.

[22] Stahl E., Praetorius F., de Oliveira Mann C.C., Hopfner K.-P., Dietz H. (2016). Impact of heterogeneity and lattice bond strength on DNA triangle crystal growth. ACS Nano.

[23] Keller A., Linko V. (2020). Challenges and perspectives of DNA nanostructures in biomedicine. Angew. Chem. Int. Ed..

[24] Jiang S., Ge Z., Mou S., Yan H., Fan C. (2021). Designer DNA nanostructures for therapeutics. Chem.

[25] Wang R., Zhang G., Liu H. (2018). DNA-templated nanofabrication. Curr. Opin. Colloid Interface Sci..

[26] Liu F., Shang Y., Wang Z., Jiao Y., Li N., Ding B. (2020). DNA origami directed fabrication of shape-controllable nanomaterials. APL Mater..

[27] Maune H.T., Han S.-P., Barish R.D., Bockrath M., Goddard W.A., Rothemund P.W.K., Winfree E. (2010). Self-assembly of carbon nanotubes into two-dimensional geometries using DNA origami templates. Nat. Nanotechnol..

[28] Linko V., Leppiniemi J., Paasonen S.-T., Hytönen V.P., Toppari J.J. (2011). Defined-size DNA triple crossover construct for molecular electronics: Modification, positioning and conductance properties. Nanotechnology.

[29] Graugnard E., Hughes W.L., Jungmann R., Kostiainen M.A., Linko V. (2017). Nanometrology and super-resolution imaging with DNA. MRS Bull..

[30] Schenkenbach M., Bauer J., Zähringer J., Selbach F., Tinnefeld P. (2020). DNA origami nanorulers and emerging reference structures. APL Mater..

[31] Kuzyk A., Jungmann R., Acuna G.P., Liu N. (2018). DNA origami route for nanophotonics. ACS Photonics.

[32] Shen B., Kostiainen M.A., Linko V. (2018). DNA origami nanophotonics and plasmonics at interfaces. Langmuir.

[33] Castro C.E., Dietz H., Högberg B. (2017). DNA origami devices for molecular-scale precision measurements. MRS Bull..

[34] Ding T., Yang J., Pan V., Zhao N., Lu Z., Ke Y., Zhang C. (2020). DNA nanotechnology assisted nanopore-based analysis. Nucleic Acids Res..

[35] Shen B., Piskunen P., Nummelin S., Liu Q., Kostiainen M.A., Linko V. (2020). Advanced DNA nanopore technologies. ACS Appl. Bio Mater..

[36] DeLuca M., Shi Z., Castro C.E., Arya G. (2020). Dynamic DNA nanotechnology: Toward functional nanoscale devices. Nanoscale Horiz..

[37] Nummelin S., Shen B., Piskunen P., Liu Q., Kostiainen M.A., Linko V. (2020). Robotic DNA nanostructures. ACS Synth. Biol..

[38] Stephanopoulos N. (2020). Hybrid nanostructures from the self-assembly of proteins and DNA. Chem.

[39] Asor R., Khaykelson D., Ben-nun-Shaul O., Levi-Kalisman Y., Oppenheim A., Raviv U. (2020). pH stability and disassembly mechanism of wild-type simian virus 40. Soft Matter.

[40] Mikkilä J., Eskelinen A.-P., Niemelä E.H., Linko V., Frilander M.J., Törmä P., Kostiainen M.A. (2014). Virus-encapsulated DNA origami nanostructures for cellular delivery. Nano Lett..

[41] Zhou K., Ke Y., Wang Q. (2018). Selective in situ assembly of viral protein onto DNA origami. J. Am. Chem. Soc..

[42] Stephanopoulos N., Liu M., Tong G.J., Li Z., Liu Y., Yan H., Francis M.B. (2010). Immobilization and one-dimensional arrangement of virus capsids with nanoscale precision using DNA origami. Nano Lett..

[43] Linko V., Mikkilä J., Kostiainen M.A., Wege C., Lomonossoff G.P. (2018). Packaging DNA origami into viral protein cages. Virus-Derived Nanoparticles for Advanced Technologies.

[44] Kopatz I., Zalk R., Levi-Kalisman Y., Zlotkin-Rivkin E., Frank G.A., Kler S. (2019). Packaging of DNA origami in viral capsids. Nanoscale.

[45] Zhou K., Zhou Y., Pan V., Wang Q., Ke Y. (2020). Programming dynamic assembly of viral proteins with DNA origami. J. Am. Chem. Soc..

[46] Perrault S.D., Shih W.M. (2014). Virus-inspired membrane encapsulation of DNA nanostructures to achieve in vivo stability. ACS Nano.

[47] Julin S., Nonappa, Shen B., Linko V., Kostiainen M.A. (2021). DNA-origami-templated growth of multilamellar lipid assemblies. Angew. Chem. Int. Ed..

[48] Linko V., Shen B., Tapio K., Toppari J.J., Kostiainen M.A., Tuukkanen S. (2015). One-step large-scale deposition of salt-free DNA origami nanostructures. Sci. Rep..

[49] Yang Y., Wang J., Shigematsu H., Xu W., Shih W.M., Rothman J.E., Lin C. (2016). Self-assembly of size-controlled liposomes on DNA nanotemplates. Nat. Chem..

[50] Zhang Z., Yang Y., Pincet F., Llaguno M.C., Lin C. (2017). Placing and shaping liposomes with reconfigurable DNA nanocages. Nat. Chem..

[51] Hernandez-Garcia A., Estrich N.A., Werten M.W.T., Van Der Maarel J.R.C., LaBean T.H., de Wolf F.A., Cohen Stuart M.A., de Vries R. (2017). Precise coating of a wide range of DNA templates by a protein polymer with a DNA binding domain. ACS Nano.

[52] Auvinen H., Zhang H., Nonappa, Kopilow A., Niemelä E.H., Nummelin S., Correia A., Santos H.A., Linko V., Kostiainen M.A. (2017). Protein coating of DNA nanostructures for enhanced stability and immunocompatibility. Adv. Healthcare Mater..

[53] Wang S.-T., Gray M.A., Xuan S., Lin Y., Byrnes J., Nguyen A.I., Todorova N., Stevens M.M., Bertozzi C.R., Zuckermann R.N. (2020). DNA origami protection and molecular interfacing through engineered sequence-defined peptoids. Proc. Natl. Acad. Sci. USA.

[54] Lacroix A., Edwardson T.G.W., Hancock M.A., Dore M.D., Sleiman H.F. (2017). Development of DNA nanostructures for high-affinity binding to human serum albumin. J. Am. Chem. Soc..

[55] Xu X., Fang S., Zhuang Y., Wu S., Pan Q., Li L., Wang X., Sun X., Liu B., Wu Y. (2019). Cationic albumin encapsulated DNA origami for enhanced cellular transfection and stability. Materials.

[56] Schaffert D.H., Okholm A.H., Sørensen R.S., Nielsen J.S., Tørring T., Rosen C.B., Kodal A.L.B., Mortensen M.R., Gothelf K.V., Kjems J. (2016). Intracellular delivery of a planar DNA origami structure by the transferrin-receptor internalization pathway. Small.

[57] Chopra A., Krishnan S., Simmel F.C. (2016). Electrotransfection of polyamine folded DNA origami structures. Nano Lett..

[58] Veneziano R., Moyer T.J., Stone M.B., Wamhoff E.-C., Read B.J., Mukherjee S., Shepherd T.R., Das J., Schief W.R., Irvine D.J. (2020). Role of nanoscale antigen organization on B-cell activation probed using DNA origami. Nat. Nanotechnol..

[59] Kwon P.S., Ren S., Kwon S.-J., Kizer M.E., Kuo L., Xie M., Zhu D., Zhou F., Zhang F., Kim D. (2020). Designer DNA architecture offers precise and multivalent spatial pattern-recognition for viral sensing and inhibition. Nat. Chem..

[60] Piskunen P., Shen B., Keller A., Toppari J.J., Kostiainen M.A., Linko V. (2021). Biotemplated lithography of inorganic nanostructures (BLIN) for versatile patterning of functional materials. ACS Appl. Nano Mater..

[61] Ramakrishnan S., Ijäs H., Linko V., Keller A. (2018). Structural stability of DNA origami nanostructures under application-specific conditions. Comput. Struct. Biotechnol. J..

[62] Bila H., Kurisinkal E.E., Bastings M.M.C. (2019). Engineering a stable future for DNA-origami as a biomaterial. Biomater. Sci..

[63] Hahn J., Wickham S.F., Shih W.M., Perrault S.D. (2014). Addressing the instability of DNA nanostructures in tissue culture. ACS Nano.

[64] Surana S., Shenoy A.R., Krishnan Y. (2015). Designing DNA nanodevices for compatibility with the immune system of higher organisms. Nat. Nanotechnol..

[65] Ponnuswamy N., Bastings M.M.C., Nathwani B., Ryu J.H., Chou L.Y.T., Vinther M., Li W.A., Anastassacos F.M., Mooney D.J., Shih W.M. (2017). Oligolysine-based coating protects DNA nanostructures from low-salt denaturation and nuclease degradation. Nat. Commun..

[66] Kielar C., Xin Y., Shen B., Kostiainen M.A., Grundmeier G., Linko V., Keller A. (2018). On the stability of DNA origami nanostructures in low-magnesium buffers. Angew. Chem. Int. Ed..

[67] Bastings M.M.C., Anastassacos F.M., Ponnuswamy N., Leifer F.G., Cuneo G., Lin C., Ingber D.E., Ryu J.H., Shih W.M. (2018). Modulation of the cellular uptake of DNA origami through control over mass and shape. Nano Lett..

[68] Ramakrishnan S., Shen B., Kostiainen M.A., Grundmeier G., Keller A., Linko V. (2019). Real-Time observation of superstructure-dependent DNA origami digestion by DNase I using high-speed atomic force microscopy. ChemBioChem.

[69] Anastassacos F.M., Zhao Z., Zeng Y., Shih W.M. (2020). Glutaraldehyde cross-linking of oligolysines coating DNA origami greatly reduces susceptibility to nuclease degradation. J. Am. Chem. Soc..

[70] Chandrasekaran A.R. (2021). Nuclease resistance of DNA nanostructures. Nat. Rev. Chem..

[71] Ijäs H., Shen B., Heuer-Jungemann A., Keller A., Kostiainen M.A., Liedl T., Ihalainen J.A., Linko V. (2021). Unraveling the interaction between doxorubicin and DNA origami nanostructures for customizable chemotherapeutic drug release. Nucleic Acids Res..

[72] Chung Y.H., Cai H., Steinmetz N.F. (2020). Viral nanoparticles for drug delivery, imaging, immunotherapy, and theranostic applications. Adv. Drug Deliv. Rev..

[73] Coleridge E.L., Dunn K.E. (2020). Assessing the cost-effectiveness of DNA origami nanostructures for targeted delivery of anti-cancer drugs to tumors. Biomed. Phys. Eng. Express..

[74] Schüller V.J., Heidegger S., Sandholzer N., Nickels P.C., Suhartha N.A., Endres S., Bourquin C., Liedl T. (2011). Cellular immunostimulation by CpG-sequence-coated DNA origami structures. ACS Nano.

[75] Liu S., Jiang Q., Zhao X., Zhao R., Wang Y., Wang Y., Liu J., Shang Y., Zhao S., Wu T. (2021). A DNA nanodevice-based vaccine for cancer immunotherapy. Nat. Mater..

